# 3*H*-1,2-benzoxathiepine 2,2-dioxides: a new class of isoform-selective carbonic anhydrase inhibitors

**DOI:** 10.1080/14756366.2017.1316720

**Published:** 2017-05-24

**Authors:** Aleksandrs Pustenko, Dmitrijs Stepanovs, Raivis Žalubovskis, Daniela Vullo, Andris Kazaks, Janis Leitans, Kaspars Tars, Claudiu T. Supuran

**Affiliations:** a Latvian Institute of Organic Synthesis, Riga, Latvia;; b Institute of Technology of Organic Chemistry, Faculty of Materials Science and Applied Chemistry, Riga Technical University, Riga, Latvia;; c Dipartimento di Chimica, Laboratorio di Chimica Bioinorganica, Polo Scientifico, Università degli Studi di Firenze, Sesto Fiorentino, Florence, Italy;; d Latvian Biomedical Research and Study Centre, Riga, Latvia;; e Faculty of Biology, Department of Molecular Biology, University of Latvia, Riga, Latvia;; f Dipartimento Neurofarba, Sezione di ScienzeFarmaceutiche e Nutraceutiche, Università degli Studi di Firenze, Sesto Fiorentino, Florence, Italy

**Keywords:** Carbonic anhydrase, sulfocoumarin, homo-sulfocoumarins, inhibitor

## Abstract

A new chemotype with carbonic anhydrase (CA, EC 4.2.1.1) inhibitory action has been discovered, the homo-sulfocoumarins (3*H*-1,2-benzoxathiepine 2,2-dioxides) which have been designed considering the (sulfo)coumarins as lead molecules. An original synthetic strategy of a panel of such derivatives led to compounds with a unique inhibitory profile and very high selectivity for the inhibition of the tumour associated (CA IX/XII) over the cytosolic (CA I/II) isoforms. Although the CA inhibition mechanism with these new compounds is unknown for the moment, we hypothesize that it may be similar to that of the sulfocoumarins, i.e. hydrolysis to the corresponding sulfonic acids which thereafter anchor to the zinc-coordinated water molecule within the enzyme active site.

## Introduction

**Figure 1.**
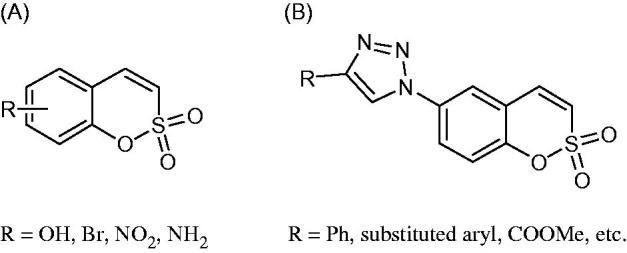
Chemical structure of sulfocoumarins **A** and **B**.

Sulfocoumarins (1,2-benzoxathiine 2,2-dioxides) such as derivatives of type **A** were discovered by our groups to act as inhibitors of the metalloenzyme carbonic anhydrase (CA, EC 4.2.1.1)^1^^,^^2^. A large series of sulfocoumarins derivatives, among which compounds of type **B**, were thereafter reported, by using click chemistry or other conventional drug design approaches (Figure 1)^3–6^.

**Scheme 1.**
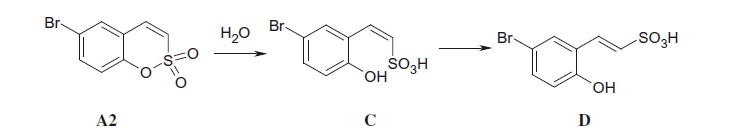
Active site, CA-mediated hydrolysis of **A2** to **D**^1^.

A salient feature of this type of CA inhibitor (CAI) was the fact that they showed a very pronounced isoform selectivity for inhibiting tumour-associated CA isoforms (CA IX and XII) over the widespread, cytosolic ones CA I and II^1–3^. This has been explained when the mechanism of CA inhibition with sulfocoumarins was elucidated, by using kinetic and X-ray crystallographic experiments^1^. Indeed, in the X-ray crystal structure of the adduct of a CA II/IX mimic complexed with the 6-bromosulfocoumarin **A2**(**A,** R = Br) (Figure 1), the 2-dihydroxy-5-bromophenyl-vinyl sulfonic acid **D** was observed within the enzyme active site, probably due to the CA-mediated hydrolysis of **A2** to the *cis*-sulfonic acid **C** which was thereafter isomerized to the more stable *trans*-derivative **D** (Scheme 1)^1^.

This inhibition mechanism is similar to the one observed earlier for coumarins^7^^,^^8^ the class of CAIs which constituted the lead compounds for the discovery of sulfocoumarins. Finding isoform-selective CAIs for the 15 different human CA isoforms is a challenging task^9^^,^^10^, but coumarins and sulfocoumarins (and several families of sulfonamides) do show such properties, which make them of great interest for the design of pharmacological agents useful as diuretics, antiglaucoma, anticonvulsant and/or antitumor drugs^9–13^.

Here, we report the homo-sulfocoumarins or 3*H*-1,2-benzoxathiepine 2,2-dioxides, which can be considered as homologs of sulfocoumarins or 1,2-benzoxathiine 2,2-dioxides^1^, where oxathiine ring was expanded by one carbon to form an oxathiepine ring. To the best of our knowledge, there is no reported method for the synthesis of 3*H*-1,2-benzoxathiepine 2,2-dioxides in the literature. The general strategy for the formation of oxathiepine ring reported in this paper involves a ruthenium-catalysed olefin metathesis as a key step.

## Materials and methods

### Chemistry

Reagents, starting materials and solvents were obtained from commercial sources and used as received. Thin-layer chromatography was performed on silica gel, spots were visualized with UV light (254 and 365 nm). Melting points were determined on an OptiMelt automated melting point system. IR spectra were measured on Shimadzu FTIR IR Prestige-21 spectrometer. NMR spectra were recorded on Varian Mercury (400 MHz) spectrometer with chemical shifts values (*δ*) in ppm relative to TMS using the residual DMSO-d_6_ signal (^1^H 2.50; 13C 39.52) or CDCl_3_ signal (^1^H 7.26; 13C 77.16) as an internal standard. HRMS data were obtained with a Q-TOF micro high resolution mass spectrometer with ESI (ESI+/ESI). Elemental analyses were performed on a CARLO ERBA ELEMENTAL ANALYZER EA 1108.

#### General procedure for the synthesis of 4-substituted 2-ethenylphenoles (2a–c)^14^

To a stirred solution of methyltriphenylphosphonium bromide (2.64 eq.) in dry THF (5 ml/1 mmol of corresponding aldehyde), was added tBuOK (2.86–3.12 eq.) in several portions over 20 min. Reaction mixture was stirred for 1 h at RT. Corresponding 2-hydroxy benzaldehyde (1 eq.) was added and stirring continued at room temperature for 24 h. Reaction mixture was diluted with CH_2_Cl_2_ (5 ml/1 mmol aldehyde). Organic layer was collected and washed with water (2 × 20 ml) and brine (2 × 20 ml), dried over Na_2_SO_4_, solvent was driven off in vacuum. The crude product was purified by column chromatography (silica gel, EtOAc/PhMe1:5).

### 2-Ethenylphenol (2a)

Compound **2a** was prepared according to the general procedure from methyltriphenylphosphonium bromide (18.88 g, 52.9 mmol), tBuOK (6.42 g, 57.2 mmol) and 2-hydroxybenzaldehyde (2.44 g, 20.0 mmol) as yellowish at room temperature melting solid (1.67 g, 70%).^1^HNMR (400 MHz, CDCl_3_) *δ* = 5.37 (dd, 1H, *J* = 11.3, 1.3 Hz), 5.42 (s, 1H), 5.76 (dd, 1H, *J* = 17.8, 1.3 Hz), 6.81 (dd, 1H, *J* = 8.1, 1.1 Hz), 6.90–6.96 (m, 1H), 6.98 (dd, 1H, *J* = 17.8, 11.3 Hz), 7.12–7.18 (m, 1H), 7.41 (dd, 1H, *J* = 7.7, 1.7 Hz).

### 4-Bromo-2-ethenylphenol (2b)

Compound **2b** was prepared according to the general procedure from methyltriphenylphosphonium bromide (13.22 g, 37.0 mmol), tBuOK (4.90 g, 43.7 mmol) and 5-bromo-2-hydroxybenzaldehyde (2.81 g, 14.0 mmol) as yellowish at room temperature melting solid (1.64 g, 59%).^1^H NMR (400 MHz, CDCl_3_) *δ* = 4.98 (s, 1H), 5.40 (dd, 1H, *J* = 11.3, 1.0 Hz), 5.74 (dd, 1H, *J* = 17.8, 1.0 Hz), 6.68 (d, 1H, *J* = 8.6 Hz), 6.85 (dd, 1H, *J* = 17.8, 8.6 Hz), 7.23 (dd, 1H, *J* = 8.6, 2.4 Hz), 7.49 (d, 1H, *J* = 2.4 Hz).

### 2-Ethenyl-4-nitrophenol (2c)

Compound **2c** was prepared according to the general procedure from methyltriphenylphosphonium bromide (28.31 g, 79.3 mmol), tBuOK (9.60 g, 85.6 mmol) and 5-nitro-2-hydroxybenzaldehyde (5 g, 30 mmol) as yellow at room temperature melting solid (3.23 g, 65%). ^1^H NMR (400 MHz, CDCl_3_) *δ* = 5.43 (dd, 1H, *J =* 11.3, 1.1 Hz), 5.87 (dd, 1H, *J* = 17.8, 1.1 Hz), 6.92–7.00 (m, 2H), 7.96 (dd, 1H, *J* = 8.9, 2.6 Hz), 8.31(d, 1H, *J* = 2.6 Hz), 8.82 (s, 1H).

### Prop-2-ene-1-sulfonyl chloride (3)^15^

To a solution of 3-bromoprop-1-ene (24.2 g, 0.20 mol) in water (140 ml) was added Na_2_SO_3_ (30 g, 0.24 mol) and the reaction mixture was refluxed overnight. After cooling to room temperature, reaction mixture was washed with Et_2_O (3 × 35 ml). Aqueous phase was concentrated. Crude white solid was dried under high vacuum at 110 °C for 4 h. To the white solid at 0 °C POCl_3_ (80 ml) was added, and mixture was refluxed for 4 h. After cooling to room temperature dry THF (60 ml) was added and reaction mixture was vigorously stirred for 10 min and filtered. Filter cake was suspended in dry THF (60 ml), suspension was vigorously stirred for 10 min and filtered. Filtrates were combined and solvent was carefully driven off on rotary evaporator. Residue was distilled in vacuum (10 mbar) and fraction with boiling point 38–42 °C was collected, to give prop-2-ene-1-sulfonil chloride (**3**) as colourless oil (18.8 g, 67%).

#### General procedure for the synthesis of 4-substituted 2-ethenyl prop-2-ene-1-sulfonates (4a–c)

To a stirred solution of corresponding 2-ethenylphenol **2** (1 eq.) in CH_2_Cl_2_ (10 ml/20 mmol phenol) at 0 °C was added prop-2-ene-1-sulfonyl chloride (**3**) (1.6 eq.) and Et_3_N (1.5 eq.). Reaction mixture was stirred overnight (20 h) at room temperature. Water (10 ml/20 mmol phenol) was added, reaction mixture was extracted with EtOAc (3 × 10 ml/20 mmol phenol), combined organic extracts were washed with brine (2 × 10 ml/20 mmol olefin), dried over Na_2_SO_4_, filtered and solvent was driven off in vacuum. The crude product was purified by column chromatography (silica gel, CH_2_Cl_2_/PhMe 3:2).

### 2-Ethenylphenyl prop-2-ene-1-sulfonate (4a)

Compound **4a** was prepared according to the general procedure from 2-ethenylphenol (**2a**) (0.50 g, 4.16 mmol), prop-2-ene-1-sulfonyl chloride (**3**) (0.94 g, 6.69 mmol) and Et_3_N (0.87 ml, 6.23 mmol) as colourless oil (0.52 g, 56%). IR (film, cm^−1^) *ν*_max_= 1368 (S=O), 1178 (S=O), 1154 (S=O); ^1^H NMR (400 MHz, CDCl_3_) *δ* = 3.96–4.00 (m, 2H), 5.37–5.41 (m, 1H), 5.48–5.54 (m, 2H), 5.79 (dd, 1H, *J* = 17.6, 0.9 Hz), 5.90–6.01 (m, 1H), 6.99 (dd, 1H, *J* = 17.6, 11.0 Hz), 7.23–7.34 (m, 2H), 7.57–7.62 (m, 1H); ^13^C NMR (100 MHz, CDCl_3_) *δ* = 55.6, 117.3, 122.8, 123.9, 125.4, 126.9, 127.4, 129.2, 130.3, 131.3, 146.5; HRMS (ESI) *m/z* [M − 1]^−^ calcd for C_11_H_11_O_3_S: 223.0429, found 223.0435.

### 4-Bromo-2-ethenylphenyl prop-2-ene-1-sulfonate (4b)

Compound **4b** was prepared according to the general procedure from 4-bromo-2-ethenylphenol (**2b**) (0.50 g, 2.51 mmol), prop-2-ene-1-sulfonyl chloride (**3**) (0.57 g, 4.05 mmol) and Et_3_N (0.52 ml, 3.76 mmol) as colourless oil (0.51 g, 67%). IR (film, cm^−1^) *ν*_max_= 1364 (S=O), 1170 (S=O), 1154 (S=O); ^1^H NMR (400 MHz, CDCl_3_) *δ* = 4.00 (dt, 2H, *J* = 7.4, 0.9 Hz), 5.46 (d, 1H, *J* = 11.0 Hz), 5.51–5.59 (m, 2H), 5.81 (d, 1H, *J* = 17.6 Hz), 5.91–6.03 (m, 1H), 6.92 (dd, 1H, *J* = 17.6, 11.0 Hz), 7.22 (d, 1H, *J =* 8.6 Hz), 7.41 (dd, 1H, *J =* 8.6, 2.4 Hz), 7.73 (d, 1H, *J =* 2.4 Hz); ^13^C NMR (100 MHz, CDCl_3_) *δ* = 55.7, 118.6, 121.0, 123.7, 124.6, 125.7, 129.2, 129.8, 132.0, 133.3, 145.3;HRMS (ESI) *m/z* [M − 1]^−^ calcd for C_11_H_10_BrO_3_S: 300.9534, found 300.9537.

### 2-Ethenyl-4-nitrophenyl prop-2-ene-1-sulfonate (4c)

Compound **4c** was prepared according to the general procedure from 2-ethenyl-4-nitrophenol (**2c**) (0.32 g, 1.94 mmol), prop-2-ene-1-sulfonyl chloride (**3**) (0.44 g, 3.13 mmol) and Et_3_N (0.41 ml, 2.96 mmol) as yellowish oil (0.30 g, 57%). IR (film, cm^−1^) *ν*_max_= 1350 (S=O), 1159 (S=O); ^1^H NMR (400 MHz, CDCl_3_) *δ* = 4.01 (dt, 2H, *J* = 7.2, 0.9 Hz), 5.54–5.63 (m, 3H), 5.93–6.05 (m, 2H), 6.99 (dd, 1H, *J* = 17.6, 11.0 Hz), 7.53 (d, 1H, *J* = 9.0 Hz), 8.16 (dd, 1H, *J* = 9.0, 2.8 Hz), 8.48 (d, 1H, *J* = 2.8 Hz); ^13^C NMR (100 MHz, CDCl_3_) *δ* = 56.3, 120.2, 122.4, 123.4, 123.8, 124.0, 126.2, 128.6, 132.8, 146.5, 150.2; HRMS (ESI) *m/z* [M − 1]^−^ calcd for C_11_H_10_NO_5_S: 268.0280, found 268.0280.

#### General procedure for the synthesis of 7-substitued 3H-1,2-benzoxathiepine 2,2-dioxides (6a–c)

To a stirred solution of corresponding 4-substituted 2-ethenyl prop-2-ene-1-sulfonate (1 eq.) in dry toluene (10 ml/0.2 g **4**), was added Ru-catalyst **5** (tricyclohexylphosphine[1,3-bis(2,4,6-trimethylphenyl)imidazol-2-ylidene][3-phenyl-1*H*-inden-1-ylidene]ruthenium(II) dichloride, CAS Nr. 254972–49-1) (0.05 eq.). Reaction mixture was stirred at 70 °C for 4 h. Solvent was driven off in vacuum and the crude product was purified by column chromatography (silica gel, Hex/EtOAc 4:1) with following re-crystallization from EtOAc/Hex. Compound **6c** was purified by column chromatography (silica gel, CH_2_Cl_2_/Hex 2:1).
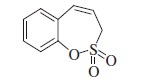


#### 3*H*-1,2-benzoxathiepine 2,2-dioxide (**6a**)

Compound **5a** was prepared according to the general procedure from 2-ethenylphenyl prop-2-ene-1-sulfonate (**4a)** (100 mg, 0.45 mmol), Ru-catalyst **5** (21 mg, 0.022 mmol) as white solid (76 mg, 87%). Mp 131–132 °C. IR (film, cm^−1^) *ν*_max_= 1369 (S=O), 1176 (S=O); ^1^H NMR (400 MHz, CDCl_3_) *δ* = 4.01 (dd, 2H, *J* = 6.3, 1.2 Hz), 5.96–6.03 (m, 1H), 6.90 (d, 1H, *J* = 10.9 Hz), 7.31–7.37 (m, 3H), 7.41–7.46 (m, 1H); ^13^C NMR (100 MHz, CDCl_3_) *δ* = 51.2, 119.5, 123.0, 127.3, 128.4, 130.6, 130.8, 132.9, 147.8;HRMS (ESI) *m/z* [M − 1]^−^ calcd for C_9_H_7_O_3_S: 195.0116, found 195.0115.
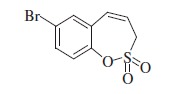


### 7-Bromo-3*H*-1,2-benzoxathiepine 2,2-dioxide (6b)

Compound **5b** was prepared according to the general procedure from 4-bromo-2-ethenylphenyl prop-2-ene-1-sulfonate (**4b**) (100 mg, 0.33 mmol), Ru-catalyst **5** (16 mg, 0.017 mmol) as yellowish solid (76 mg, 84%). Mp 129.3–130.3 °C. IR (film, cm^−1^) *ν*_max_= 1360 (S=O), 1170 (S=O), 1154 (S=O); ^1^H NMR (400 MHz, CDCl_3_) *δ* = 4.03 (dd, 2H, *J* = 6.3, 0.9 Hz), 5.99–6.06 (m, 1H), 6.81 (d, 1H, *J* = 11.0 Hz), 7.22 (d, 1H, *J* = 8.6 Hz), 7.47 (d, 1H, *J* = 2.4 Hz), 7.54 (dd, 1H, *J* = 8.6, 2.4 Hz); ^13^C NMR (100 MHz, CDCl_3_) *δ* = 51.4, 120.5, 120.9, 124.7, 130.2, 131.6, 133.5, 133.6, 146.7; Anal. Calcd for C_9_H_7_BrO_3_S (275.12): C 39.29, H 2.56, found C 39.19, H 2.59.
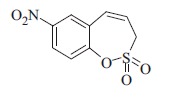


### 7-Nitro-3*H*-1,2-benzoxathiepine 2,2-dioxide (6c)

Compound **5c** was prepared according to the general procedure from 2-ethenyl-4-nitrophenyl prop-2-ene-1-sulfonate (**4c**) (100 mg, 0.37 mmol), catalyst **5** (18 mg, 0.019 mmol) as yellowish solid (86 mg, 96%). Mp 130–131 °C. IR (film, cm^−1^) *ν*_max_= 1375 (S=O), 1351 (S=O), 1170 (S=O), 1161 (S=O); ^1^H NMR (400 MHz, CDCl_3_) *δ* = 4.18 (dd, 2H, *J* = 5.8, 1.2 Hz), 6.05–6.12 (m, 1H), 6.89 (d, 1H, *J* = 11.3 Hz), 7.48 (d, 1H, *J* = 8.9 Hz),8.24 (d, 1H, *J* = 2.6 Hz), 8.28 (dd, 1H, *J* = 8.9, 2.6 Hz); ^13^C NMR (100 MHz, CDCl_3_) *δ* = 52.4, 121.6, 124.3, 125.6, 126.8, 129.4, 130.8, 151.3; Anal. Calcd for C_9_H_7_NO_5_S (241.22): C 44.81, H 2.92, N 5.81, found C 44.70, H 2.95, N 5.79.
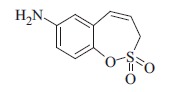


### 7-Amino-3*H*-1,2-benzoxathiepine 2,2-dioxide (7)

To a solution of 7-nitro-3*H*-1,2-benzoxathiepine 2,2-dioxide (**6c**) (250 mg, 1.04 mmol) in EtOH (4.3 ml) and H_2_O (2.8 ml) AcOH (0.06 ml, 1.04 mmol) was added following by iron powder (350 mg, 6.27 mmol) at room temperature. Resulting suspension was stirred at 75 °C for 1 h. It was cooled to room temperature, EtOAc (50 ml) was added and washed with sat. aq. NaHCO_3_ (5 × 30 ml). Organic layer was dried over Na_2_SO_4_ and concentrated in vacuum. Re-crystallized of the crude product from EtOAc/Hex afforded **7** (220 mg, 98%) as yellowish solid. Mp 170–171 °C. IR (film, cm^−1^) *ν*_max_=3465 (N–H), 3382 (N–H), 1358 (S=O), 1163 (S=O); ^1^H NMR (400 MHz, CDCl_3_) *δ* = 3.72–3.85 (br s,2H), 3.92 (dd, 2H, *J* = 6.3, 1.0 Hz), 5.93–6.00 (m, 1H), 6.53 (d, 1H, *J* = 2.9 Hz), 6.68 (dd, 1H, *J* = 8.8, 2.6 Hz), 6.80 (d, 1H, *J* = 10.6 Hz), 7.12 (d, 1H, *J* = 8.8 Hz); ^13^C NMR (100 MHz, CDCl_3_) *δ* = 50.5, 115.0, 116.8, 119.8, 123.8, 133.4, 140.4, 145.5; HRMS (ESI) *m/z* [M + H]^+^ calcd for C_9_H_10_NO_3_S: 212.0381, found 212.0364.
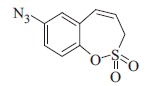


### 7-Azido-3*H*-1,2-benzoxathiepine 2,2-dioxide (8)

To a solution of -7-amino-3*H*-1,2-benzoxathiepine 2,2-dioxide (**7**) (220 mg, 1.03 mmol) in trifluoroacetic acid (1.3 ml) at 0 °C, slowly was added NaNO_2_ (80 mg, 1.12 mmol). After 30 min stirring at 0 °C, solution of NaN_3_ (67 mg, 1.03 mmol) in water (3 ml) was added. Mixture was stirring at 0 °C for1 h. Collection of solid precipitate and drying in vacuum afforded **8** (170 mg, 69%) as brown solid. IR (film, cm^−1^) *ν*_max_= 2116 (N_3_), 1374 (S=O), 1369 (S=O), 1167 (S=O); ^1^H NMR (400 MHz, CDCl_3_) *δ* = 4.01 (dd, 2H, *J* = 6.3, 1.2 Hz), 5.99–6.07 (m, 1H), 6.83 (d, 1H, *J* = 10.9 Hz), 6.94 (d, 1H, *J* = 2.8 Hz), 7.06 (dd, 1H, *J* = 8.9, 2.8 Hz), 7.32 (d, 1H, *J* = 8.9 Hz); ^13^C NMR (100 MHz, CDCl_3_) *δ* = 51.2, 120.5, 120.8, 120.9, 124.5, 129.8, 132.0, 139.2, 144.5.

#### General procedure for the synthesis of 1,4-disubstitutedtriazolyl compound (9–17)

To a solution of corresponding alkyne (1 eq.) in tBuOH/H_2_O 1:1 mixture (10 ml)7-azido-3*H*-1,2-benzoxathiepine 2,2-dioxide (**8**) (1 eq.), CuSO_4_·5H_2_O (2 eq.) and sodium ascorbate (4 eq.) were added and reaction mixture was stirred at room temperature for 10 min. AcOH (19–21 eq.) was added and mixture was stirred for additional 30 min. Solvent was driven off in vacuum and the crude product was purified by reversed phase chromatography (C-18, H_2_O–MeCN gradient MeCN 10–90%).
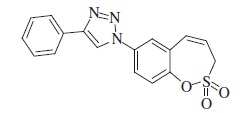


### 1-(2,2-Dioxido-3*H*-1,2-benzoxathiepin-7-yl)-4-phenyl-1*H*-1,2,3-triazole (9)

Compound **9** was prepared according to the general procedure from phenylacetylene (13 mg, 0.13 mmol), azide **8** (30 mg, 0.13 mmol), CuSO_4_·5H_2_O (65 mg, 0.26 mmol)_,_ sodium ascorbate (103 mg, 0.52 mmol), AcOH (0.14 ml, 2.45 mmol) as white solid (41 mg, 95%). Mp 203–204 °C. IR (KBr, cm^−1^) *ν*_max_=1368 (S=O), 1171 (S=O); ^1^H NMR (400 MHz, DMSO-d_6_) *δ* = 4.61 (dd, 2H, *J* = 5.9, 1.2 Hz), 6.09–6.16 (m, 1H), 7.02 (d, 1H, *J* = 11.3 Hz), 7.37–7.43 (m, 1H), 7.48–7.54 (m, 2H), 7.63 (d, 1H, *J* = 8.8 Hz), 7.92–7.97 (m, 2H), 8.04 (dd, 1H, *J* = 8.8, 2.6 Hz), 8.13 (d, 1H, *J* = 2.6 Hz), 9.35 (s, 1H); ^13^C NMR (100 MHz, DMSO-d_6_) *δ* = 51.7, 119.9, 121.6, 122.1, 122.7, 124.0, 125.3, 128.4, 129.1, 129.6, 130.0, 130.1, 135.0, 146.3, 147.5; HRMS (ESI) *m/z* [M + H]^+^ calcd for C_17_H_14_N_3_O_3_S: 340.0756, found 340.0755.
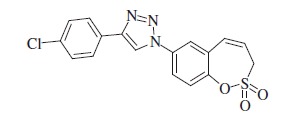


### 4-(4-Chlorophenyl)-1–(2,2-dioxido-3*H*-1,2-benzoxathiepin-7-yl)-1*H*-1,2,3-triazole (10)

Compound **10** was prepared according to the general procedure from 1-chloro-4-ethynylbenzene (17 mg, 0.12 mmol), azide **8** (29 mg, 0.12 mmol), CuSO_4_·5H_2_O (61 mg, 0.24 mmol)_,_ sodium ascorbate (97 mg, 0.49 mmol), AcOH (0.13 ml, 2.27 mmol) as yellowish solid (34 mg, 74%). Mp 191–192 °C. IR (KBr, cm^−1^) *ν*_max_=1369 (S=O), 1356 (S=O), 1168 (S=O); ^1^H NMR (400 MHz, DMSO-d_6_) *δ* = 4.61 (dd, 2H, *J* = 5.9, 1.2 Hz), 6.09–6.16 (m, 1H), 7.01 (d, 1H, *J* = 11.5 Hz), 7.55–7.61 (m, 2H), 7.63 (d, 1H, *J* = 8.9 Hz), 7.92–7.98 (m, 2H), 8.02 (dd, 1H, *J =* 8.9, 2.7 Hz), 8.11 (d, 1H, *J* = 2.7 Hz), 9.38 (s, 1H); ^13^C NMR (100 MHz, DMSO-d_6_) *δ* = 51.7, 120.3, 121.6, 122.1, 122.7, 124.1, 127.0, 129.0, 129.1, 129.6, 130.1, 132.8, 135.0, 146.3, 146.4; HRMS (ESI) *m/z* [M + H]^+^ calcd for C_17_H_13_ClN_3_O_3_S: 374.0366, found 374.0366.
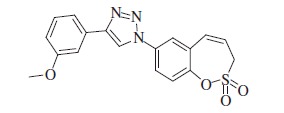


### 1-(2,2-Dioxido-3*H*-1,2-benzoxathiepin-7-yl)-4-(3-methoxyphenyl)-1*H*-1,2,3-triazole (11)

Compound **11** was prepared according to the general procedure from 3-ethynylanisole (17 mg, 0.13 mmol), azide **8** (30 mg, 0.13 mmol), CuSO_4_·5H_2_O (63 mg, 0.25 mmol)_,_ sodium ascorbate (100 mg, 0.50 mmol), AcOH (0.14 ml, 2.45 mmol) as yellowish solid (24 mg, 51%). Mp210–211 °C.IR (KBr, cm^−1^) *ν*_max_=1372 (S=O), 1162 (S=O); ^1^H NMR (400 MHz, DMSO-d_6_) *δ* = 3.84 (s, 3H), 4.61 (dd, 2H, *J* = 5.8, 1.2 Hz), 6.09–6.16 (m, 1H), 6.94–6.99 (m, 1H), 7.02 (d, 1H, *J* = 11.5 Hz), 7.39–7.45 (m, 1H), 7.48–7.55 (m, 2H), 7.63 (d, 1H, *J* = 8.9 Hz), 8.03 (dd, 1H, *J* = 8.9, 2.7 Hz), 8.12 (d, 1H, *J* = 2.7 Hz), 9.36 (s, 1H); ^13^C NMR (100 MHz, DMSO-d_6_) *δ* = 51.7, 55.2, 110.6, 114.1, 117.6, 120.1, 121.6, 122.1, 122.6, 124.0, 129.6, 130.1,130.2, 131.4, 135.0, 146.3, 147.4, 159.8; HRMS (ESI) *m/z* [M + H]^+^ calcd for C_18_H_16_N_3_O_4_S: 370.0862, found 370.0876.
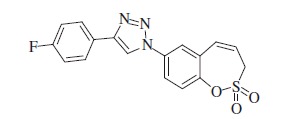


### 1-(2,2-Dioxido-3*H*-1,2-benzoxathiepin-7-yl)-4–(4-fluorophenyl)-1*H*-1,2,3-triazole (12)

Compound **12** was prepared according to the general procedure from 1-ethynyl-4-fluorobenzene (30 mg, 0.25 mmol), azide **8** (60 mg, 0.25 mmol), CuSO_4_·5H_2_O (126 mg, 0.50 mmol), sodium ascorbate (200 mg, 1.02 mmol), AcOH (0.28 ml, 5.05 mmol) as yellowish solid (60 mg, 66%). Mp 200–201 °C. IR (KBr, cm^−1^) *ν*_max_=1369 (S=O), 1167 (S=O); ^1^H NMR (400 MHz, DMSO-d_6_) *δ* = 4.61 (d, 2H, *J* = 5.4 Hz), 6.07–6.17 (m, 1H), 7.01 (d, 1H, *J* = 11.3 Hz), 7.30–7.71 (m, 2H), 7.63 (d, 1H, *J* = 8.8 Hz), 7.94–8.05 (m, 3H), 8.11 (s, 1H), 9.34 (s, 1H); ^13^C NMR (100 MHz, DMSO-d_6_) *δ* = 51.7, 116.1 (d, *J =* 21.9 Hz), 119.9, 121.6, 122.1, 122.7, 124.1, 126.6, 127.4 (d, *J =* 8.3 Hz), 129.7, 130.1, 135.0, 146.3, 146.6, 162.1 (d, *J =* 245.3 Hz); HRMS (ESI) *m/z* [M + H]^+^ calcd for C_17_H_13_FN_3_O_3_S: 358.0662, found 358.0656.
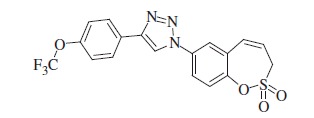


### 1-(2,2-Dioxido-3*H*-1,2-benzoxathiepin-7-yl)-4-[4-(trifluorometoxy)phenyl]-1*H*-1,2,3-triazole (13)

Compound **13** was prepared according to the general procedure from 4-(trifluoromethoxy) phenylacetylene (40 mg, 0.21 mmol), azide **8** (50 mg, 0.21 mmol), CuSO_4_·5H_2_O (105 mg, 0.42 mmol), sodium ascorbate (167 mg, 0.84 mmol), AcOH (0.23 ml, 4.02 mmol) as yellowish solid (74 mg, 83%). Mp 168–169 °C. IR (film, cm^−1^) *ν*_max_= 1357 (S=O), 1166 (S=O); ^1^H NMR (400 MHz, CDCl_3_) *δ* = 4.13 (dd, 2H, *J* = 6.0, 1.1Hz), 6.06–6.13 (m, 1H), 6.93 (d, 1H, *J* = 11.3 Hz), 7.30–7.35 (m, 2H), 7.51 (d, 1H, *J* = 8.8 Hz), 7.79 (dd, 1H, *J* = 8.8, 2.5 Hz), 7.85 (d, 1H, *J* = 2.5 Hz), 7.91–7.98 (m, 2H), 8.25 (s, 1H); ^13^C NMR (100 MHz, CDCl_3_) *δ* = 51.8, 120.6 (q, *J* = 257.9 Hz), 121.4, 121.7, 122.1, 122.9, 124.7, 127.5, 128.8, 130.0, 131.5, 135.6, 147.3, 149.5, 149.6; HRMS (ESI) *m/z* [M + H]^+^ calcd for C_18_H_13_F_3_N_3_O_4_S: 424.0579, found 424.0553.
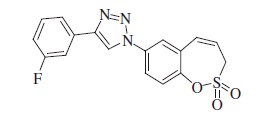


### 1-(2,2-Dioxido-3*H*-1,2-benzoxathiepin-7-yl)-4–(3-fluorophenyl)-1*H*-1,2,3-triazole (14)

Compound **14** was prepared according to the general procedure from 1-ethynyl-3-fluorobenzene (25 mg, 0.21 mmol), azide **8** (50 mg, 0.21 mmol), CuSO_4_·5H_2_O (105 mg, 0.42 mmol), sodium ascorbate (166 mg, 0.84 mmol), AcOH (0.25 ml, 4.37 mmol) as brownish solid (56 mg, 74%). Mp 188–189 °C. IR (KBr, cm^−1^) *ν*_max_=1354 (S=O), 1175 (S=O); ^1^H NMR (400 MHz, DMSO-d_6_) *δ* = 4.62 (dd, 2H, *J* = 6.0, 1.3 Hz), 6.09–6.16 (m, 1H), 7.01 (d, 1H, *J* = 11.6 Hz), 7.20–7.26 (m, 1H), 7.52–7.60 (m, 1H), 7.64 (d, 1H, *J* = 8.8 Hz), 7.70–7.75 (m, 1H), 7.77–7.81 (m, 1H), 8.02 (dd, 1H, *J* = 8.9, 2.7 Hz), 8.10 (d, 1H, *J* = 2.7 Hz), 9.42 (s, 1H); ^13^C NMR (100 MHz, DMSO-d_6_) *δ* = 51.7, 111.9 (d, *J* = 23.0 Hz), 115.1 (d, *J* = 20.8 Hz), 120.7, 121.3 (d, *J* = 2.5 Hz), 121.6, 122.1, 122.7, 124.1, 129.6, 130.0, 131.2 (d, *J* = 8.7 Hz), 132.4 (d, *J* = 8.4 Hz), 134.9, 146.3, 146.4, 162.6 (d, *J* = 243.5 Hz); HRMS (ESI) *m/z*[M + H]^+^ calcd for C_17_H_13_FN_3_O_3_S: 358.0662, found 358.0667.
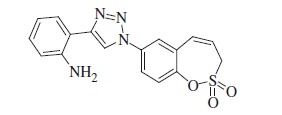


### 2-[1-(2,2-Dioxido-3*H*-1,2-benzoxathiepin-7-yl)-1*H*-1,2,3-triazol-4-yl]aniline (15)

Compound **15** was prepared according to the general procedure from 2-ethynylaniline (25 mg, 0.21 mmol), azide **8** (50 mg, 0.21 mmol), CuSO_4_·5H_2_O (105 mg, 0.42 mmol), sodium ascorbate (166 mg, 0.84 mmol), AcOH (0.25 ml, 4.37 mmol) as yellowish solid (43 mg, 57%). Mp 190–191 °C. IR (film, cm^−1^) *ν*_max_=3430 (N–H), 3364 (N–H), 1365 (S=O), 1358 (S=O), 1167 (S=O), 1163 (S=O); ^1^H NMR (400 MHz, DMSO-d_6_) *δ* = 4.61 (dd, 2H, *J* = 6.0, 1.2 Hz), 6.09–6.16 (m, 1H), 6.49–6.85 (m, 2H), 7.01 (d, 1H, *J* = 11.3 Hz), 7.10–7.18 (m, 1H), 7.59–7.66 (m, 2H), 8.08 (dd, 1H, *J* = 8.9, 2.4 Hz), 8.16 (d, 1H, *J* = 2.4 Hz), 9.26 (s, 1H); ^13^C NMR (100 MHz, DMSO-d_6_) *δ* = 51.7, 112.1, 115.9, 116.1, 119.8, 121.8, 122.1, 122.8, 124.0, 127.9, 129.0, 129.6, 130.1, 135.0, 145.8, 146.3, 148.1; HRMS (ESI) *m/z*[M + H]^+^ calcd for C_17_H_15_N_4_O_3_S: 355.0865, found 355.0869.
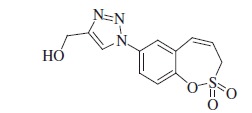


### [1-(2,2-dioxido-3*H*-1,2-benzoxathiepin-7-yl)-1*H*-1,2,3-triazol-4-yl]methanol (16)

Compound **16** was prepared according to the general procedure from propargyl alcohol (0.012 ml, 0.21 mmol), azide **8** (50 mg, 0.21 mmol), CuSO_4_·5H_2_O (105 mg, 0.42 mmol), sodium ascorbate (166 mg, 0.84 mmol), AcOH (0.25 ml, 4.37 mmol) as white solid (50 mg, 81%). Mp 144–145 °C. IR (KBr, cm^−1^) *ν*_max_= 1374 (S=O), 1167 (S=O); ^1^H NMR (400 MHz, DMSO-d_6_) *δ* = 4.59 (d, 2H, *J* = 5.7 Hz), 4.62 (s, 2H), 6.05–6.13 (m, 1H), 6.98 (d, 1H, *J=* 11.5 Hz), 7.56 (d, 1H, *J* = 8.9 Hz), 7.99 (dd, 1H, *J* = 8.9, 2.6 Hz), 8.09 (d, 1H, *J* = 2.6 Hz), 8.74 (s, 1H); ^13^C NMR (100 MHz, DMSO-d_6_) *δ* = 51.8, 54.9, 121.3, 121.5, 121.9, 122.6, 123.9, 129.5, 130.1, 135.1, 146.1, 149.4; HRMS (ESI) *m/z* [M + H]^+^ calcd for C_12_H_12_N_3_O_4_S: 294.0549, found 294.0553.
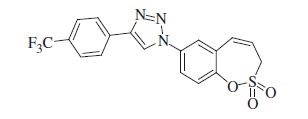


### 4-(2,2-Dioxido-3*H*-1,2-benzoxathiepin-7-yl)-1-[4-(trifluoromethyl)phenyl]-1*H*-1,2,3-triazole (17)

Compound **17** was prepared according to the general procedure from 4-(trifluoromethyl)phenylacetylene (36 mg, 0.21 mmol), azide **8** (50 mg, 0.21 mmol), CuSO_4_·5H_2_O (105 mg, 0.42 mmol), sodium ascorbate (166 mg; 0.84 mmol), AcOH (0.25 ml, 4.37 mmol) as yellowish solid (73 mg, 85%). Mp 192–193 °C.IR (KBr, cm^−1^) *ν*_max_= 1358 (S=O), 1328 (S=O), 1174 (S=O), 1166 (S=O); ^1^H NMR (400 MHz, DMSO-d_6_) *δ* = 4.62 (dd, 2H, *J* = 5.9, 1.0 Hz), 6.09–6.16 (m, 1H), 7.01 (d, 1H, *J* = 11.5 Hz), 7.64 (d, 1H, *J* = 8.8 Hz), 7.86–7.91 (m, 2H), 8.04 (dd, 1H, *J* = 8.8, 2.7 Hz), 8.13 (d, 1H, *J* = 2.7 Hz), 8.13–8.18 (m, 2H), 9.52 (s, 1H); ^13^C NMR (100 MHz, DMSO-d_6_) *δ* = 51.7, 121.2, 121.7, 122.1, 122.8, 124.1, 124.2(q, *J* = 272.0 Hz), 125.8, 126.1 (q, *J* = 3.8 Hz), 128.4 (q, *J* = 32.0 Hz), 129.6, 130.0, 134.0, 134.9, 146.0, 146.4; HRMS (ESI) *m/z* [M + H]^+^ calcd for C_18_H_13_F_3_N_3_O_3_S: 408.0630, found 408.0626.

## CA inhibition assay

An SX.18 MV-R Applied Photophysics (Oxford, UK) stopped-flow instrument has been used to assay the catalytic/inhibition of various CA isozymes^16^. Phenol Red (at a concentration of 0.2 mM) has been used as indicator, working at the absorbance maximum of 557 nm, with 10 mM Hepes (pH 7.4) as buffer, 0.1 M Na_2_SO_4_ or NaClO_4_ (for maintaining constant the ionic strength; these anions are not inhibitory in the used concentration),^17^ following the CA-catalysed CO_2_ hydration reaction for a period of 5–10 s. Saturated CO_2_ solutions in water at 25 °C were used as substrate. Stock solutions of inhibitors were prepared at a concentration of 10 mM (in DMSO-water 1:1, v/v) and dilutions up to 1 nM done with the assay buffer mentioned above. At least seven different inhibitor concentrations have been used for measuring the inhibition constant. Inhibitor and enzyme solutions were preincubated together for 6 h at 4 °C prior to assay, in order to allow for the formation of the E–I complex. Triplicate experiments were done for each inhibitor concentration, and the values reported throughout the paper are the mean of such results. The inhibition constants were obtained by non-linear least-squares methods using the Cheng–Prusoff equation, as reported earlier^17^, and represent the mean from at least three different determinations. All CA isozymes used here were recombinant proteins obtained as reported earlier by our group^18^.

## X-ray structure determination

X-Ray diffraction data for compound **6c** were collected using a *NoniusKappaCCD* diffractometre (MoKα radiation, *λ* = 0.71073 Å), equipped with low temperature *Oxford CryosystemsCryostream Plus* device (Delft, the Netherlands). Data were collected using *KappaCCD* Server Software, cell refined by *SCALEPACK*^19^, data reduction performed by *DENZO*^20^ and *SCALEPACK*^19^, structures solved by direct method using *SIR2004* and refined by *SHELXL97*^21^ as implemented in the program package *WinGX*^22^. Software used to prepare CIF file was SHELXL97^21^ and graphics–*ORTEP3*^22^.

*Crystal data for***6c**: C_9_H_7_NO_5_S (*M* = 241.22), monoclinic, *P*2_1_/*a*, *a* = 7.3194(3), *b* = 14.9000(7) and *c* = 18.3387(8) Å, *β* = 101.325(1)°, *V* = 1961.06(15) Å^3^, *T* = 173(2) K, *Z* = 2, *Z'* = 1, μ(MoKα) = 0.34 mm^−1^, 9545 reflections measured, 2150 independent reflections (*R*_int_ = 0.083), *R*_1_(obs) = 0.058, *wR*1(obs) = 0.1500, *R*_1_(all) = 0.1893, *wR*1(all) = 0.1096, *S* = 0.94.

CCDC 1526002 contains the supplementary crystallographic data for this paper. These data can be obtained free of charge from The Cambridge Crystallographic Data Centre *via*http://www.ccdc.cam.ac.uk.

## Results and discussion

### Chemistry

**Scheme 2.**
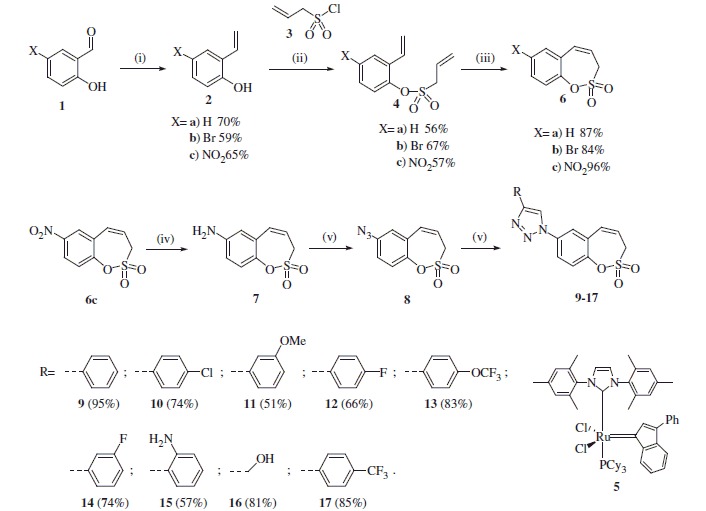
Reagents and conditions: (i) MePPh_3_Br, tBuOK, THF, RT, 24 h; (ii) NEt_3_, CH_2_Cl_2_, RT, 20 h; (iii) **5**, toluene, 70 °C, 4 h; (iv) Fe, AcOH, EtOH, H_2_O, 70 °C, 1 h, 98%; (v) 1) NaNO_2_, H_2_O, TFA, 2) NaN_3_, H_2_O, 69%; (vi) alkyne, tBuOH/H_2_O (1:1), CuSO_4_, sodium ascorbate, acetic acid, 30 min.

The synthesis of homo-sulfocoumarins began with a Wittig reaction in which salicylic aldehydes **1** were converted to the corresponding mono-olefins **2a–c** in good yields (Scheme 2). Treatment of compounds **2a–c** with allyl sulfonyl chloride (**3**) provided *bis*-olefins **4a–c** as the key intermediates, again in good yields (see Experimental for details). In the next step, olefin metathesis with the commercially available Ru-catalyst **5** was used, in which *bis*-olefins **4a–c** were converted to 3*H*-1,2-benzoxathiepine 2,2-dioxides **6a–b** in 84–96% yields. To obtain a series of 7-substituted homo-sulfocoumarins, the synthesis of 1,4-triazolyl derivatives **9–17** was thereafter performed. For this purpose, 7-nitro derivative **6c** was reduced by elemental iron to the corresponding amine **7** in nearly quantitative yield. Further diazotation of amine **7** followed by *in situ* treatment with sodium azide afforded the azide **8**. Treatment of azide **8** with alkynes under click chemistry condition provides a series of 1,4-triazolyl homo-sulfocoumarins **9–17** in good to excellent yields (see Experimental for details).

**Figure 2.**
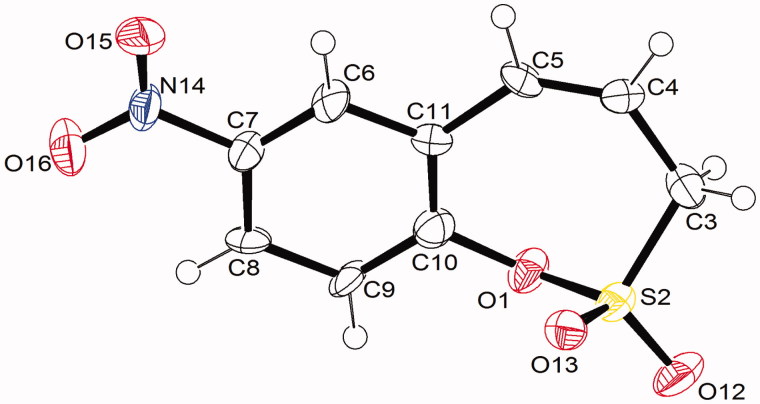
Single-crystal X-ray structure of **6c** (CCDC deposition number 1526002). Thermal ellipsoids are drawn at the 50% probability level (see Experimental for details).

The structures of all synthesized 3*H*-1,2-benzoxathiepine 2,2-dioxides **6–17** were fully supported by ^1^H, ^13^C NMR and IR spectroscopy, MS or elemental analysis. Additionally, the final unequivocal identification of the scaffold of 3*H*-1,2-benzoxathiepine 2,2-dioxide was established by a single-crystal X-ray structure for compound **6c**, shown in Figure 2.

### Carbonic anhydrase inhibition

All the synthesized derivatives **6c–17** were evaluated for their efficacy in inhibiting four relevant CA isoforms, i.e. hCA I, II, IX and XII, by using the stopped flow carbon dioxide hydrase assay^16^, in comparison to the sulphonamide acetazolamide (**AAZ**, 5-acetamido-1,3,4-thiadiazole-2-sulfonamde) as a standard CAI.

Data of Table 1 show that the cytosolic isoforms hCA I and II (widely distributed enzymes, with important physiological roles in many tissues)^9^^,^^10^ were generally not inhibited by the investigated homo-sulfocoumarins, up to 50 μM concentration of inhibitors in the assay system. Only one derivative, **13**, showed a moderate inhibitory profile against hCA II, with an inhibition constant of 5.77 μM.

**Table 1.** CA inhibition data against isoforms hCA I, II, IX and XII with homo-sulfocoumarins **6–17** and acetazolamide (**AAZ**) as standard, by a stopped-flow CO_2_ hydrase assay^14^.

	K_I_ (μM)^a^			
Compound	hCA I	hCA II	hCA IX	hCA XII
**6c**	>50	>50	0.027	0.64
**7**	>50	>50	3.57	>50
**9**	>50	>50	1.71	>50
**10**	>50	>50	3.59	>50
**11**	>50	>50	2.56	>50
**12**	>50	>50	1.75	>50
**13**	>50	5.77	0.34	1.72
**14**	>50	>50	1.15	>50
**15**	>50	>50	0.46	2.32
**16**	>50	>50	0.87	>50
**17**	>50	>50	0.43	>50
**AAZ**	0.25	0.012	0.025	0.006

a Errors in the range of ±5% of the reported values, from three different assays.

The tumour associated isoform hCA IX, a validated drug target for antitumor/antimetastatic agents^23^^,^^24^, was on the other hand effectively inhibited by the investigated homo-sulfocoumarins, with K_I_s ranging between 27 nM and 3.59 μM (Table 1). The structure activity relationship (SAR) was very interesting, as the best inhibitor (**6c**) incorporated a compact, powerful electron attracting moiety (NO_2_) whereas the remaining derivatives, incorporating substituted 1,2,3-triazole moieties in position 7 of the homo-sulfocoumarin ring were less effective hCA IX inhibitors. Four submicromolar hCA IX inhibitors were however detected apart **6c**, derivatives **13, 15, 16** and **17**, which incorporate either the compact hydroxymethyl group at the triazole fragment of the molecule, or substituted phenyls with 4-trifluoromethoxy-, 2-amino-, or 4-trifluoromethyl substituents on the aryl fragment. These derivatives showed K_I_s ranging between 0.34 and 0.87 μM. The remaining homo-sulfocoumarins were low micromolar hCA IX inhibitors.

The SAR for inhibition of the second tumour-associated isoform, hCA XII, was more complex compared to what discussed above for hCA IX (Table 1). Thus, 8 out of 11 derivatives were inactive (K_I_s > 50 μM) whereas the remaining ones, **6c, 13** and **15**, inhibited hCA XII with K_I_s in the range of 0.64–2.32 μM.

This inhibition profile is rather similar to the one of sulfocoumarins^1–6^ and coumarins^7^^,^^8^, which are generally selective inhibitors for the tumour-associated over the cytosolic isoforms. However, some homo-sulfocoumarins showed a very specific, and unique up until now inhibition profile among all classes of CAIs known to date^9^^,^^10^, as they are highly selective for hCA IX over hCA I, II and XII (e.g. **7–12, 14, 16** and **17**).

In conclusion, we report here a new chemotype with effective and isoform-selective CAIs, the homo-sulfocoumarins, which show a unique inhibition profile for the tumour-associated CA isoforms hCA IX (and XII) over the cytosolic ones. Although the CA inhibition mechanism with these new compounds is unknown for the moment, we hypothesize that it may be similar to that of the sulfocoumarins, i.e. hydrolysis to the corresponding sulfonic acids which thereafter anchor to the zinc-coordinated water molecule within the enzyme active site.
